# Hyperphosphatemia-related biochemical profiles and clinical correlates in patients receiving maintenance hemodialysis: a single-center retrospective cross-sectional study

**DOI:** 10.3389/fmed.2026.1910971

**Published:** 2026-10-28

**Authors:** Lei Zhang, Licun Zhang

**Affiliations:** Tianjin Baodi Hospital, Tianjin, China

**Keywords:** biochemical patterns, blood urea nitrogen, chronic kidney disease–mineral and bone disorder, hyperphosphatemia, maintenance hemodialysis

## Abstract

**Background:**

Hyperphosphatemia is common in maintenance hemodialysis (MHD) and often coexists with other abnormalities of chronic kidney disease–mineral and bone disorder (CKD-MBD). We described predefined biochemical patterns related to hyperphosphatemia and examined their clinical correlates.

**Methods:**

This single-center retrospective cross-sectional study screened 589 patients receiving hemodialysis between March 2023 to March 2026; 254 patients comprised the final complete-case analytical cohort. Four prespecified threshold-based biochemical patterns were defined using serum phosphorus, parathyroid hormone (PTH), and alkaline phosphatase (ALP). Univariable and fixed multivariable logistic regression were used to examine factors associated with hyperphosphatemia. Restricted cubic splines were used to assess the functional forms of BUN, uric acid, and creatinine. Sensitivity analyses and 1,000 bootstrap resamples were performed to examine the robustness of the adjusted estimates.

**Results:**

Hyperphosphatemia was present in 182 patients (71.7%). The four patterns comprised 72 (28.3%) non-hyperphosphatemia, 106 (41.7%) HP/PTH−, 61 (24.0%) HP/PTH+/ALP−, and 15 (5.9%) HP/PTH+/ALP+. BUN, uric acid, and creatinine were higher in the hyperphosphatemic groups, whereas Kt/V was similar. After adjustment, creatinine was positively associated with hyperphosphatemia (OR 1.27 per 100 μmol/L, 95% CI 1.07–1.50, *P* = 0.005). Uric acid showed a nonlinear association (overall *P* = 0.005, *P* for nonlinearity=0.004), although estimates at the upper end of its distribution were less precise. The BUN association was borderline (OR 1.33 per 5 mmol/L, 95% CI 1.00–1.78, *P* = 0.054) and weakened with the stricter phosphorus cutoff.

**Conclusion:**

Predefined biochemical patterns captured heterogeneity in hyperphosphatemia among patients receiving MHD. Creatinine remained associated with hyperphosphatemia, uric acid showed a nonlinear but imprecise high-range association, and BUN was less consistent. These findings may help frame future studies of phosphate control using routinely available laboratory data.

## 1 Introduction

Chronic kidney disease-mineral and bone disorder (CKD-MBD) is still a serious problem for patients on maintenance hemodialysis (MHD), and hyperphosphatemia is one of the most common biochemical abnormalities. The current KDIGO guidelines recommend that phosphate, calcium and parathyroid hormone (PTH) be assessed together, and alkaline phosphatase (ALP) can be used to determine whether there is increased bone turnover (1). Recent KDIGO discussions have added to the demand for personalised CKD-MBD care by indicating that mineral-bone and cardiovascular disorders do not always occur together in the same individuals (2).

Large dialysis cohorts have shown that poor control of phosphorus and PTH is common and clinically relevant. In the international Dialysis Outcomes and Practice Patterns Study (DOPPS), abnormalities in mineral metabolism were frequent and were associated with adverse outcomes (3, 4). Similar difficulties have been reported in Chinese hemodialysis practice, where hyperphosphatemia and secondary hyperparathyroidism remain common despite widespread use of CKD-MBD therapies (5). Phosphate control is also shaped by factors that are not captured by phosphorus alone, including dietary protein and phosphorus intake, residual kidney function, dialysis removal, and treatment adherence. The observed relationship between hyperphosphatemia, PTH, and dietary protein intake further illustrates this complexity (6).

Much of the available literature has focused on individual CKD-MBD markers, outcome associations, or the efficacy of phosphate-lowering treatment (3–7). Less attention has been paid to the way routinely measured biochemical abnormalities coexist within the same patient and whether simple, predefined patterns are accompanied by differences in other clinical or dialysis-related features. This question is relevant to routine care, but it requires cautious interpretation. Threshold-based groups are useful for description, not proof of distinct biological phenotypes, and treatment exposure in cross-sectional data may largely reflect disease severity and prescribing decisions rather than treatment effects. We therefore used a prespecified, threshold-based framework to describe hyperphosphatemia-related biochemical patterns in an MHD cohort, based on serum phosphorus, PTH, and ALP. The study examined how these patterns were distributed and how they differed with respect to clinical, biochemical, dialysis-related, and treatment characteristics. We also assessed cross-sectional associations between selected non-defining clinical variables and hyperphosphatemia, allowing for nonlinear relationships where appropriate and examining the stability of the multivariable findings in sensitivity and bootstrap analyses. The aim was descriptive and hypothesis-generating rather than to establish a new CKD-MBD phenotype system or a predictive model.

## 2 Methods

### 2.1 Study design and participants

This was a single-center, retrospective, cross-sectional study conducted at the Blood Purification Center of Baodi Hospital of Tianjin Medical University. Patients receiving hemodialysis between March 2023 to March 2026 were screened from the center's clinical and dialysis records.

Initial eligibility required an age of ≥18 years and maintenance hemodialysis (MHD) for at least 6 months. Patients meeting these criteria were then assessed for availability of the clinical and laboratory variables required for the prespecified analyses. A complete-case approach was used. Patients with missing data for any required variable were excluded, and no missing values were imputed. Among the 483 patients who met the initial age and dialysis-vintage criteria, 178 were excluded because one or more required clinical or laboratory variables were unavailable. These patients were not retained in the analytical dataset. When the data were subsequently re-audited, the source records required to reconstruct variable-specific missingness or to compare these excluded patients with the final analytical cohort were no longer retrievable from the original clinical and laboratory systems. This left 305 patients with complete required data. Of these, 51 were subsequently excluded because they met one or more prespecified clinical exclusion criteria. These criteria included malignant disease; hematopoietic dysfunction, hemolytic disease, or another hematologic disorder likely to materially affect hematologic or iron-related measurements; severe infection, marked malnutrition, gastrointestinal bleeding, surgery, or blood transfusion within the preceding 3 months; or transfer to another hemodialysis center, conversion to peritoneal dialysis, kidney transplantation, death, or another documented reason that precluded inclusion in the final study cohort. The final analytical cohort therefore comprised 254 patients (Figure 1). No prospective sample-size calculation was performed because the study was based on an existing clinical population and all potentially eligible patients within the study period were screened. Given the size of the final cohort, the multivariable analysis was deliberately limited to a fixed set of clinically relevant covariates rather than allowing model composition to depend on univariable statistical significance. The study was approved by the Medical Ethics Committee of Tianjin Baodi Hospital (approval no. BDYYLL202626) and was conducted in accordance with the Declaration of Helsinki. The requirement for written informed consent was waived because of the retrospective use of routinely collected, anonymized clinical data.

**Figure 1:**
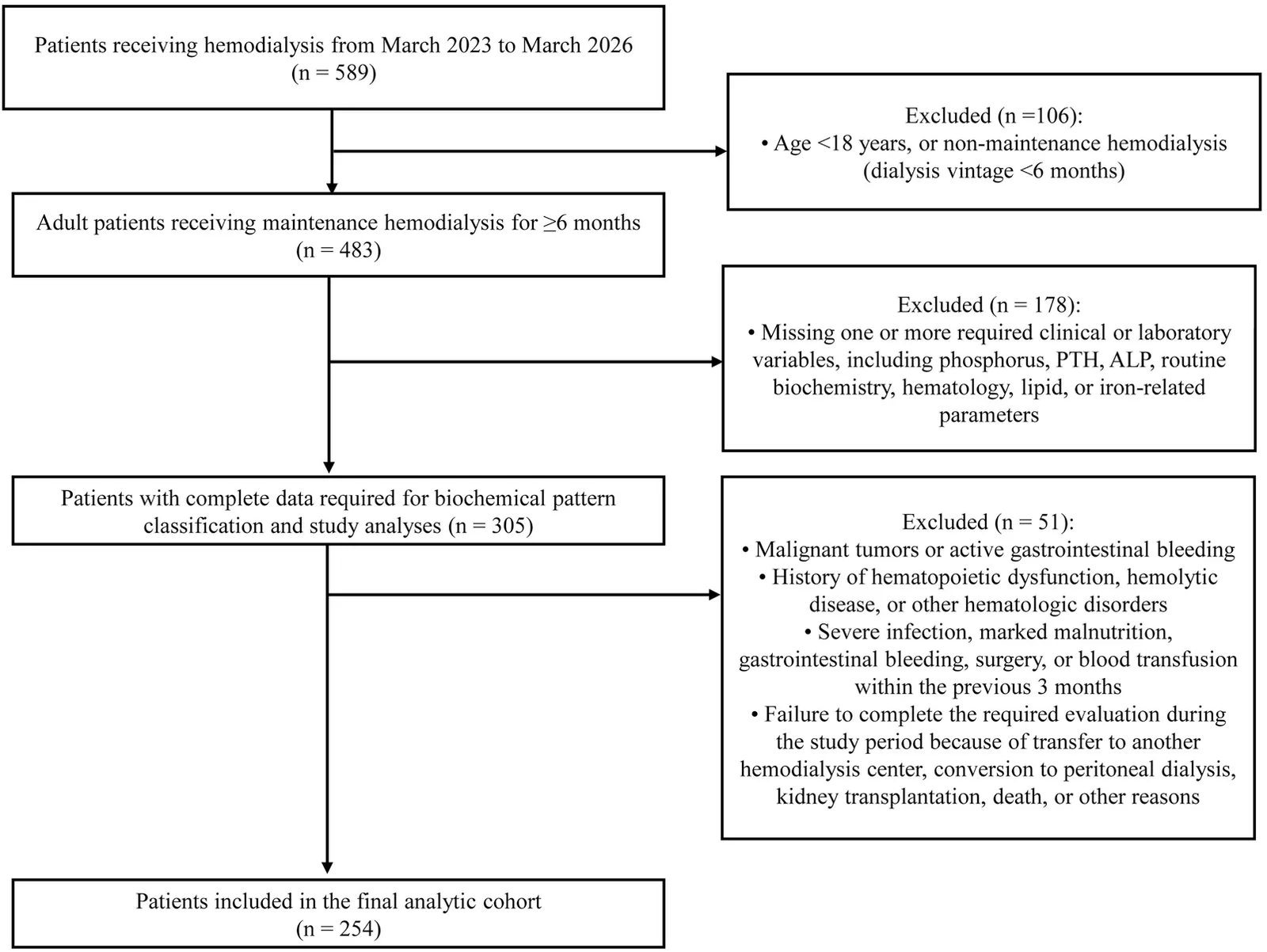
Flowchart of participant selection.

### 2.2 Data sources, index assessment, and study variables

Data were retrospectively obtained from the hospital electronic medical record system, laboratory database, and dialysis records. Demographic and clinical variables included age, sex, dialysis vintage, and primary kidney disease. Primary kidney disease was grouped as primary glomerular disease, diabetic nephropathy, hypertensive kidney damage, or other causes according to the primary renal diagnosis recorded in the medical record.

For each patient, the first available routine biochemical and CKD-MBD assessment during the study period that satisfied the prespecified data requirements was selected as the index assessment. Serum phosphorus was used as the anchor measurement. Phosphorus, alkaline phosphatase (ALP), blood urea nitrogen (BUN), creatinine, and uric acid were preferentially obtained from the same biochemical blood draw. When ALP was not available on the anchor date, the closest measurement within ±7 days was used. Parathyroid hormone (PTH) was preferentially obtained on the same date as serum phosphorus; if unavailable, the closest PTH measurement within ±30 days was used, provided that it was obtained using the same laboratory platform. Measurements outside these windows were treated as missing. Blood sampling was intended to occur before dialysis as part of routine clinical assessment. A sample was considered verifiably predialysis only when the laboratory information system timestamp preceded the documented start time of the dialysis session. When this relationship could not be established from the available records, sampling timing was considered uncertain.

Laboratory variables included hemoglobin, albumin, BUN, uric acid, creatinine, serum iron, ferritin, transferrin saturation, calcium, phosphorus, PTH, ALP, triglycerides, total cholesterol, low-density lipoprotein cholesterol (LDL-C), high-density lipoprotein cholesterol (HDL-C), and carbon dioxide combining power. Dialysis-related variables included Kt/V, dialysis frequency, duration of each dialysis session, and dialysate calcium concentration. Treatment exposure was obtained from medication records and coded as use or non-use of a phosphate binder, active vitamin D therapy, and a calcimimetic. These variables were used only to describe CKD-MBD management across the predefined biochemical patterns and were not interpreted as treatment effects. Information on dose, treatment duration, adherence, and longitudinal treatment changes was not consistently available. Because treatment is commonly initiated or intensified in response to existing CKD-MBD abnormalities, treatment exposure was not included in the primary regression models to reduce the risk of confounding by indication. When treatment status was unavailable, percentages were calculated from the available medication records without imputation.

### 2.3 Definition of hyperphosphatemia and predefined biochemical patterns

Hyperphosphatemia was defined *a priori* as a serum phosphorus concentration >1.78 mmol/L. This cutoff corresponds to 5.5 mg/dL, the upper boundary of the serum phosphorus range historically recommended by the KDOQI guideline for patients receiving dialysis (8). Current KDIGO guidance does not specify a single fixed phosphorus target and instead recommends treatment based on persistently or progressively elevated serum phosphate. In the present study, 1.78 mmol/L was used as an operational threshold for cross-sectional classification rather than as a contemporary treatment target.

A PTH concentration >300 pg/mL was used to identify greater parathyroid activation within the hyperphosphatemic group. This threshold was based on historical KDOQI guidance for dialysis patients, in which 150–300 pg/mL was used as the target range (8). It was applied only for construction of the predefined biochemical patterns and was not considered a universal diagnostic threshold for secondary hyperparathyroidism. Current KDIGO recommendations interpret PTH in CKD G5D in relation to the assay-specific upper reference limit rather than using a single absolute cutoff (1).

For serum alkaline phosphatase (ALP), a value >125 U/L was used to distinguish the two PTH-elevated hyperphosphatemic groups. This threshold corresponds to the upper reference limit reported for adult Chinese men in a large multicenter study of liver-function reference intervals, in which the ALP reference interval was 45–125 U/L for men; the corresponding intervals differed among women according to age (9). Because ALP reference intervals vary by sex and age, 125 U/L was not treated as a universal adult upper reference limit. The threshold was used only as a pragmatic cutoff for descriptive stratification and not as a diagnostic criterion for renal osteodystrophy or high-turnover bone disease. Using these thresholds, four mutually exclusive biochemical patterns were defined. Patients with serum phosphorus ≤1.78 mmol/L were classified as non-hyperphosphatemia (NH). Those with phosphorus >1.78 mmol/L and PTH ≤300 pg/mL were classified as HP/PTH−. Patients with phosphorus >1.78 mmol/L and PTH >300 pg/mL were further divided according to ALP: ALP ≤125 U/L defined HP/PTH+/ALP−, whereas ALP >125 U/L defined HP/PTH+/ALP+. These categories were specified before the comparative analyses and were treated as threshold-based biochemical patterns rather than data-derived clusters, validated CKD-MBD phenotypes, or diagnostic categories of bone turnover. Phosphorus, PTH, and ALP were retained in descriptive summaries to show the laboratory distributions underlying the classification, but differences in these defining markers were not interpreted as independent evidence of biological separation between groups.

### 2.4 Statistical analysis

All analyses were performed in R version 4.5.2. Continuous demographic and laboratory variables were summarized as median and interquartile range (IQR), whereas categorical and discretely recorded dialysis variables were summarized as number and percentage. Differences across the four predefined biochemical patterns were assessed with the Kruskal–Wallis test for continuous variables and Pearson's chi-square or Fisher's exact test for categorical variables, as appropriate. For sparse contingency tables larger than 2 × 2, Fisher's exact test was implemented using Monte Carlo simulation with 200,000 replicates. Pairwise comparisons of BUN, uric acid, creatinine, and Kt/V were performed with Dunn's test after the overall Kruskal–Wallis test, with Holm adjustment for multiple comparisons. The distributions of the four patterns were also examined descriptively across sex, primary kidney disease, and dialysis-vintage categories; no interaction analyses were performed.

Hyperphosphatemia, defined as serum phosphorus >1.78 mmol/L, was treated as the binary outcome in the association analyses. Univariable logistic regression was applied to age, sex, dialysis vintage, primary kidney disease, hemoglobin, albumin, BUN, uric acid, creatinine, serum iron, ferritin, transferrin saturation, calcium, triglycerides, total cholesterol, LDL-C, HDL-C, carbon dioxide combining power, Kt/V, dialysis frequency, dialysis-session duration, and dialysate calcium concentration. Phosphorus, PTH, and ALP were not considered candidate correlates because they were used in defining the outcome or biochemical patterns. Treatment exposure was also excluded from the regression analyses because its cross-sectional association with biochemical abnormalities is particularly susceptible to confounding by indication. Age, dialysis vintage, hemoglobin, albumin, BUN, uric acid, creatinine, serum iron, ferritin, transferrin saturation, calcium, and Kt/V were expressed per 10 years, 12 months, 10 g/L, 5 g/L, 5 mmol/L, 100 μmol/L, 100 μmol/L, 5 μmol/L, 100 ng/mL, 5 percentage points, 0.1 mmol/L, and 0.1 unit, respectively. Lipid measures and carbon dioxide combining power were analyzed per 1-unit increase. Male sex, primary glomerular disease, and the lowest observed category of each dialysis-related categorical variable served as reference groups.

The multivariable logistic regression model used a fixed adjustment set comprising age, sex, dialysis vintage, primary kidney disease, BUN, uric acid, creatinine, and Kt/V. These variables were retained to provide adjustment for basic demographic and renal-disease characteristics while accounting for nitrogenous solute burden, renal clearance-related measures, and dialysis adequacy. The adjustment set was kept fixed across the primary and sensitivity models and was not determined by univariable *P* values. No stepwise or other data-driven variable-selection procedure was used. Adjusted odds ratios (ORs) and 95% confidence intervals (CIs) were reported. Because uric acid was represented by restricted cubic spline terms in the final model, multicollinearity was assessed in a corresponding linearized model in which uric acid was entered as a single linear term. Adjusted generalized variance inflation factors (GVIFs) from this linearized model were used for the collinearity assessment.

The functional forms of BUN, uric acid, and creatinine were examined with three-knot restricted cubic splines, using the 10th, 50th, and 90th percentiles as knot locations. The spline analyses were used to assess whether the association of each marker with hyperphosphatemia departed materially from linearity. BUN and creatinine were retained as linear terms, whereas uric acid was modeled with a restricted cubic spline using knots at 254.3, 387.0, and 517.7 μmol/L. Overall and nonlinear spline effects were evaluated with likelihood-ratio tests. Because a single OR does not adequately represent a nonlinear association, the uric acid effect was additionally summarized as adjusted ORs at the 10th and 90th percentiles relative to the median. Overall *P* values for multi-level categorical variables were obtained from likelihood-ratio tests; *P* values for individual regression coefficients were based on Wald tests.

Two sensitivity analyses repeated the same multivariable model specification. The first used a stricter definition of hyperphosphatemia (serum phosphorus >2.10 mmol/L). The second excluded the three records flagged during data-quality review. The uric acid spline specification and knot locations were kept unchanged. The purpose of these analyses was to examine whether the main adjusted associations were sensitive to the outcome threshold or to exclusion of the QC-flagged records. Model robustness was further examined using 1,000 nonparametric case-resampling bootstrap replicates with the full model refitted in each replicate and no variable selection. Bootstrap 95% CIs were percentile based. Bias was assessed on the log-OR scale as the difference between the mean bootstrap estimate and the corresponding estimate from the original model. Bootstrap results were used as a robustness check rather than as evidence of predictive performance. The analytical cohort was restricted to complete cases for variables required for biochemical classification and the primary association analyses. Treatment-exposure variables were not part of this complete-case requirement; where treatment information was unavailable, percentages were calculated from available records without imputation. All statistical tests were two-sided. A *P* value < 0.05 was considered statistically significant; for *post hoc* pairwise comparisons, inference was based on Holm-adjusted *P* values. Given the cross-sectional design, regression estimates were interpreted as associations rather than causal effects or measures of predictive performance.

## 3 Results

### 3.1 Study population and distribution of predefined biochemical patterns

A total of 589 patients receiving hemodialysis were screened between March 2023 to March 2026. After the initial eligibility assessment, 483 patients remained; 178 were excluded because one or more required clinical or laboratory variables were unavailable. Among the 305 patients with complete required data, a further 51 met the prespecified clinical exclusion criteria, leaving 254 patients in the final analytical cohort (Figure 1). Hyperphosphatemia was present in 182 of the 254 patients (71.7%). According to the predefined threshold-based classification, 72 patients (28.3%) were classified as non-hyperphosphatemia (NH), 106 (41.7%) as HP/PTH−, 61 (24.0%) as HP/PTH+/ALP−, and 15 (5.9%) as HP/PTH+/ALP + (Figure 2). HP/PTH− was the most frequently observed pattern, whereas HP/PTH+/ALP + accounted for only a small proportion of the cohort. The distribution of the four biochemical patterns did not differ significantly by sex (*P* = 0.849), primary kidney disease (*P* = 0.275), or dialysis vintage (<36, 36–59, and ≥60 months; *P* = 0.733) (Supplementary Table S1). HP/PTH− remained the largest category within each of these strata. Because some cells, particularly those involving the HP/PTH+/ALP + group, contained few patients, these subgroup distributions were interpreted descriptively rather than as evidence of differential pattern prevalence across clinical strata.

**Figure 2:**
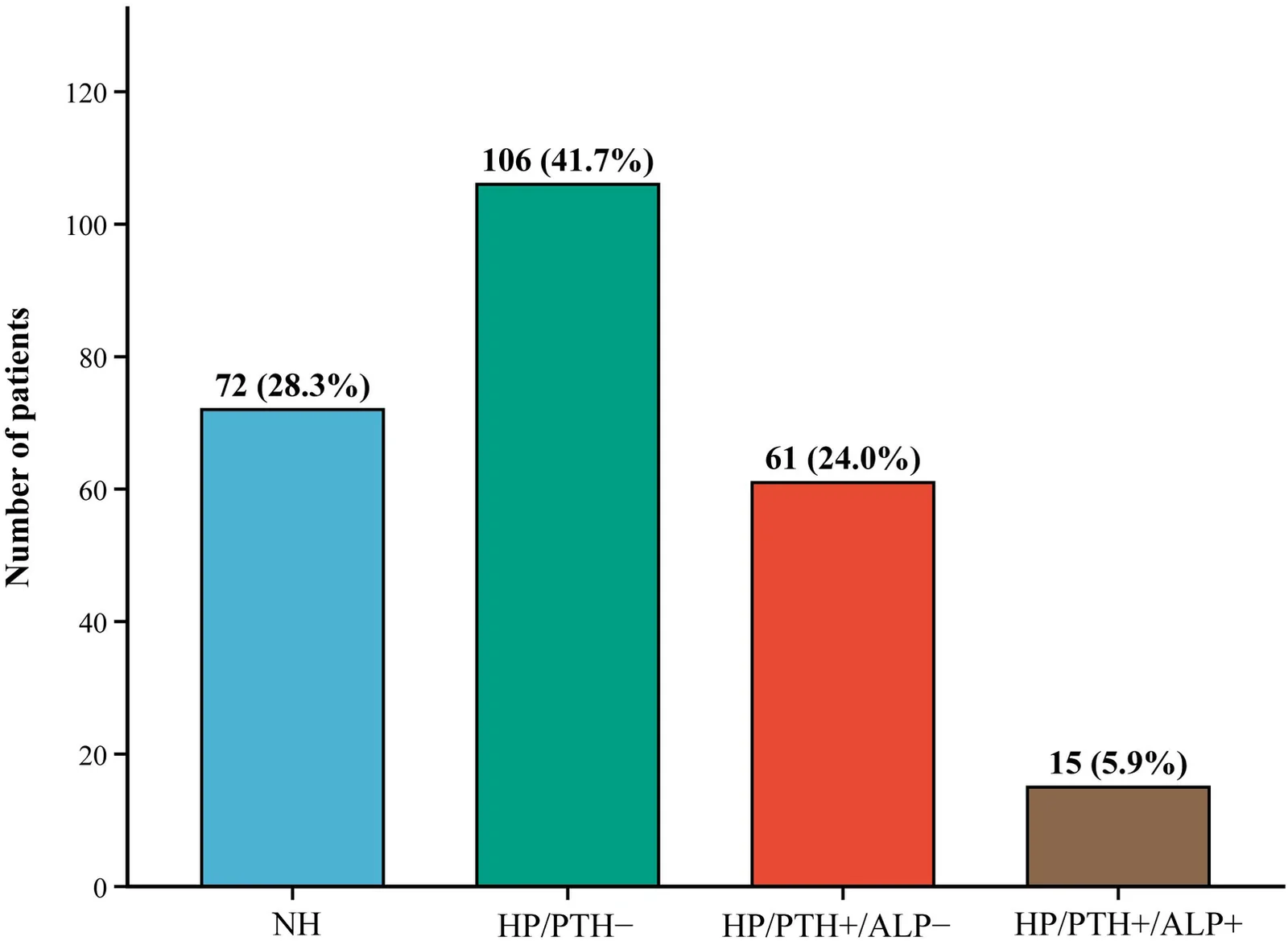
Distribution of predefined hyperphosphatemia-related biochemical patterns in the study population. Values above the bars indicate the number and percentage of patients in each pattern. NH, non-hyperphosphatemia; HP/PTH−, hyperphosphatemia without elevated parathyroid hormone; HP/PTH+/ALP−, hyperphosphatemia with elevated parathyroid hormone and alkaline phosphatase ≤125 U/L; HP/PTH+/ALP+, hyperphosphatemia with elevated parathyroid hormone and alkaline phosphatase >125 U/L.

### 3.2 Clinical, biochemical, and treatment characteristics across predefined biochemical patterns

Baseline demographic and dialysis-related characteristics were generally comparable across the four predefined biochemical patterns (Table 1). There were no clear between-group differences in age, sex, dialysis vintage, primary kidney disease, hemoglobin, albumin, iron-status measures, calcium, lipid parameters, carbon dioxide combining power, Kt/V, dialysis frequency, dialysis-session duration, or dialysate calcium concentration (*P* > 0.05). By contrast, BUN, uric acid, and creatinine differed across the four groups (*P* < 0.001; Figure 3). Median BUN was 23.30 mmol/L in the NH group and 28.42, 27.25, and 29.03 mmol/L in the HP/PTH−, HP/PTH+/ALP−, and HP/PTH+/ALP + groups, respectively. Compared with NH, BUN was higher in each hyperphosphatemic group after Holm-adjusted Dunn testing (*P* < 0.001, *P* = 0.005, and *P* = 0.009, respectively). Uric acid showed a similar pattern, with medians of 350.5, 395.0, 420.0, and 404.0 μmol/L across the four groups; the corresponding comparisons with NH were *P* = 0.016, *P* = 0.001, and *P* = 0.030. Creatinine was also higher in the three hyperphosphatemic groups than in NH (*P* < 0.001, *P* = 0.005, and *P* = 0.028). Kt/V did not differ across the groups (*P* = 0.885).

**Table 1:** Clinical, biochemical, dialysis-related, and treatment characteristics across predefined hyperphosphatemia-related biochemical patterns.

Characteristic	Overall *N* = 254	NH *N* = 72	HP-PTH− *N* = 106	HP-PTH + *N* = 61	HP-PTH+/ALP + *N* = 15	*P* value
Demographic and clinical characteristics						
Age, years	54.0 (44.0, 63.0)	56.5 (48.0, 67.5)	52.5 (44.0, 62.0)	54.0 (43.0, 63.0)	51.0 (31.0, 60.0)	0.144
Sex						0.849
Male	150 (59.1%)	40 (55.6%)	64 (60.4%)	36 (59.0%)	10 (66.7%)	
Female	104 (40.9%)	32 (44.4%)	42 (39.6%)	25 (41.0%)	5 (33.3%)	
Dialysis vintage, months	57.5 (32.0, 109.0)	59.5 (34.0, 110.0)	49.0 (27.0, 104.0)	62.0 (29.0, 133.0)	60.0 (46.0, 78.0)	0.733
Primary kidney disease						0.275
Primary glomerular disease	122 (48.0%)	34 (47.2%)	49 (46.2%)	30 (49.2%)	9 (60.0%)	
Diabetic nephropathy	55 (21.7%)	19 (26.4%)	24 (22.6%)	10 (16.4%)	2 (13.3%)	
Hypertensive kidney damage	16 (6.3%)	3 (4.2%)	8 (7.5%)	2 (3.3%)	3 (20.0%)	
Other causes	61 (24.0%)	16 (22.2%)	25 (23.6%)	19 (31.1%)	1 (6.7%)	
Laboratory characteristics						
Hemoglobin, g/L	111.0 (101.0, 121.0)	109.0 (100.0, 116.5)	112.5 (101.0, 122.0)	113.0 (100.0, 123.0)	109.0 (99.0, 122.0)	0.350
Albumin, g/L	42.80 (41.10, 44.70)	42.65 (39.95, 44.85)	42.95 (41.60, 44.80)	42.10 (41.30, 44.40)	42.80 (42.20, 46.10)	0.298
Blood urea nitrogen, mmol/L	27.11 (22.32, 31.13)	23.30 (18.99, 27.90)	28.42 (24.45, 31.84)	27.25 (22.72, 32.49)	29.03 (23.12, 37.19)	<0.001
Uric acid, μmol/L	387.0 (316.0, 451.0)	350.5 (301.5, 407.0)	395.0 (316.0, 460.0)	420.0 (340.0, 468.0)	404.0 (335.0, 594.0)	<0.001
Creatinine, μmol/L	1,034.5 (856.0, 1,201.0)	904.5 (715.0, 1,072.5)	1,121.0 (927.0, 1,288.0)	1,050.0 (891.0, 1,198.0)	1,036.0 (955.0, 1,296.0)	<0.001
Serum iron, μmol/L	11.30 (9.20, 14.00)	10.75 (8.50, 13.90)	11.30 (9.30, 14.00)	12.00 (9.30, 14.20)	9.50 (7.60, 13.00)	0.258
Ferritin, ng/mL	138.0 (68.1, 275.0)	149.5 (78.7, 265.5)	114.5 (69.7, 257.0)	135.0 (54.5, 364.0)	185.0 (32.7, 292.0)	0.822
Transferrin saturation, %	23.45 (18.92, 29.58)	22.61 (18.65, 29.62)	23.05 (19.29, 30.85)	24.90 (21.35, 29.57)	20.26 (13.57, 27.18)	0.305
Calcium, mmol/L	2.25 (2.12, 2.38)	2.24 (2.10, 2.38)	2.27 (2.12, 2.37)	2.25 (2.14, 2.37)	2.25 (2.05, 2.42)	0.927
Phosphorus, mmol/L	2.07 (1.74, 2.41)	1.54 (1.32, 1.69)	2.24 (2.00, 2.55)	2.31 (2.07, 2.61)	2.10 (1.92, 2.51)	—
PTH, pg/mL	236.4 (129.0, 402.5)	179.0 (96.1, 292.0)	156.4 (101.0, 233.0)	463.0 (375.0, 566.0)	836.0 (637.0, 1,419.0)	—
ALP, U/L	79.5 (59.0, 106.0)	86.0 (61.5, 110.5)	68.0 (53.0, 90.0)	82.0 (63.0, 106.0)	148.0 (142.0, 196.0)	—
Triglyceride, mmol/L	1.50 (1.07, 2.31)	1.50 (1.16, 2.41)	1.53 (1.02, 2.63)	1.40 (1.19, 2.09)	1.68 (1.15, 2.27)	0.914
Total cholesterol, mmol/L	3.74 (3.12, 4.49)	3.85 (3.03, 4.74)	3.52 (3.06, 4.47)	3.77 (3.37, 4.30)	4.18 (3.25, 4.60)	0.542
LDL-C, mmol/L	2.00 (1.50, 2.59)	2.07 (1.36, 2.73)	1.82 (1.40, 2.47)	2.02 (1.79, 2.52)	2.55 (1.26, 2.87)	0.388
HDL-C, mmol/L	0.91 (0.75, 1.13)	0.94 (0.80, 1.16)	0.88 (0.73, 1.08)	0.94 (0.76, 1.18)	0.84 (0.68, 1.05)	0.257
CO2 combining power, mmol/L	17.80 (16.10, 19.50)	18.30 (16.60, 19.75)	17.25 (16.10, 19.10)	17.50 (15.60, 19.80)	17.90 (15.00, 19.00)	0.214
Dialysis-related characteristics						
Kt/V	1.33 (1.22, 1.48)	1.36 (1.22, 1.49)	1.33 (1.21, 1.48)	1.35 (1.22, 1.46)	1.35 (1.15, 1.51)	0.885
Dialysis frequency, times/week						0.537
2	15 (5.9%)	2 (2.8%)	8 (7.5%)	4 (6.6%)	1 (6.7%)	
3	239 (94.1%)	70 (97.2%)	98 (92.5%)	57 (93.4%)	14 (93.3%)	
Dialysis duration, h						0.230
3	1 (0.4%)	0 (0.0%)	0 (0.0%)	1 (1.6%)	0 (0.0%)	
3.5	14 (5.5%)	3 (4.2%)	4 (3.8%)	5 (8.2%)	2 (13.3%)	
4	239 (94.1%)	69 (95.8%)	102 (96.2%)	55 (90.2%)	13 (86.7%)	
Dialysate calcium, mmol/L						0.119
1.25	13 (5.1%)	2 (2.8%)	10 (9.4%)	1 (1.6%)	0 (0.0%)	
1.50	241 (94.9%)	70 (97.2%)	96 (90.6%)	60 (98.4%)	15 (100.0%)	
Treatment exposure						
Phosphate binder use	193 (76.0%)	14 (19.4%)	104 (98.1%)	60 (98.4%)	15 (100.0%)	<0.001
Active vitamin D use	238 (93.7%)	58 (80.6%)	105 (99.1%)	60 (98.4%)	15 (100.0%)	<0.001
Calcimimetic use	221 (87.4%)	58 (80.6%)	94 (89.5%)	54 (88.5%)	15 (100.0%)	0.153

Data are presented as median (interquartile range) for continuous variables and *n* (%) for categorical variables. Percentages were calculated using available observations. *P* values were calculated using the Kruskal–Wallis test for continuous variables and the Pearson chi-square test or Fisher’s exact test for categorical variables, as appropriate. For sparse contingency tables larger than 2 × 2, Fisher’s exact test was estimated using Monte Carlo simulation (200,000 replicates). Phosphorus, PTH, and ALP were used to define the predefined biochemical patterns; formal between-group hypothesis testing was therefore not performed for these variables.

Treatment exposure is presented descriptively and should not be interpreted as evidence of treatment efficacy or harm because of potential confounding by indication. One observation for calcimimetic use was missing; percentages were calculated using available observations.

NH, non-hyperphosphatemia; HP-PTH−, hyperphosphatemia without elevated PTH; HP-PTH+, hyperphosphatemia with elevated PTH and ALP <=125 U/L; HP-PTH+/ALP+, hyperphosphatemia with elevated PTH and elevated ALP; PTH, parathyroid hormone; ALP, alkaline phosphatase; TSAT, transferrin saturation; LDL-C, low-density lipoprotein cholesterol; HDL-C, high-density lipoprotein cholesterol.

**Figure 3:**
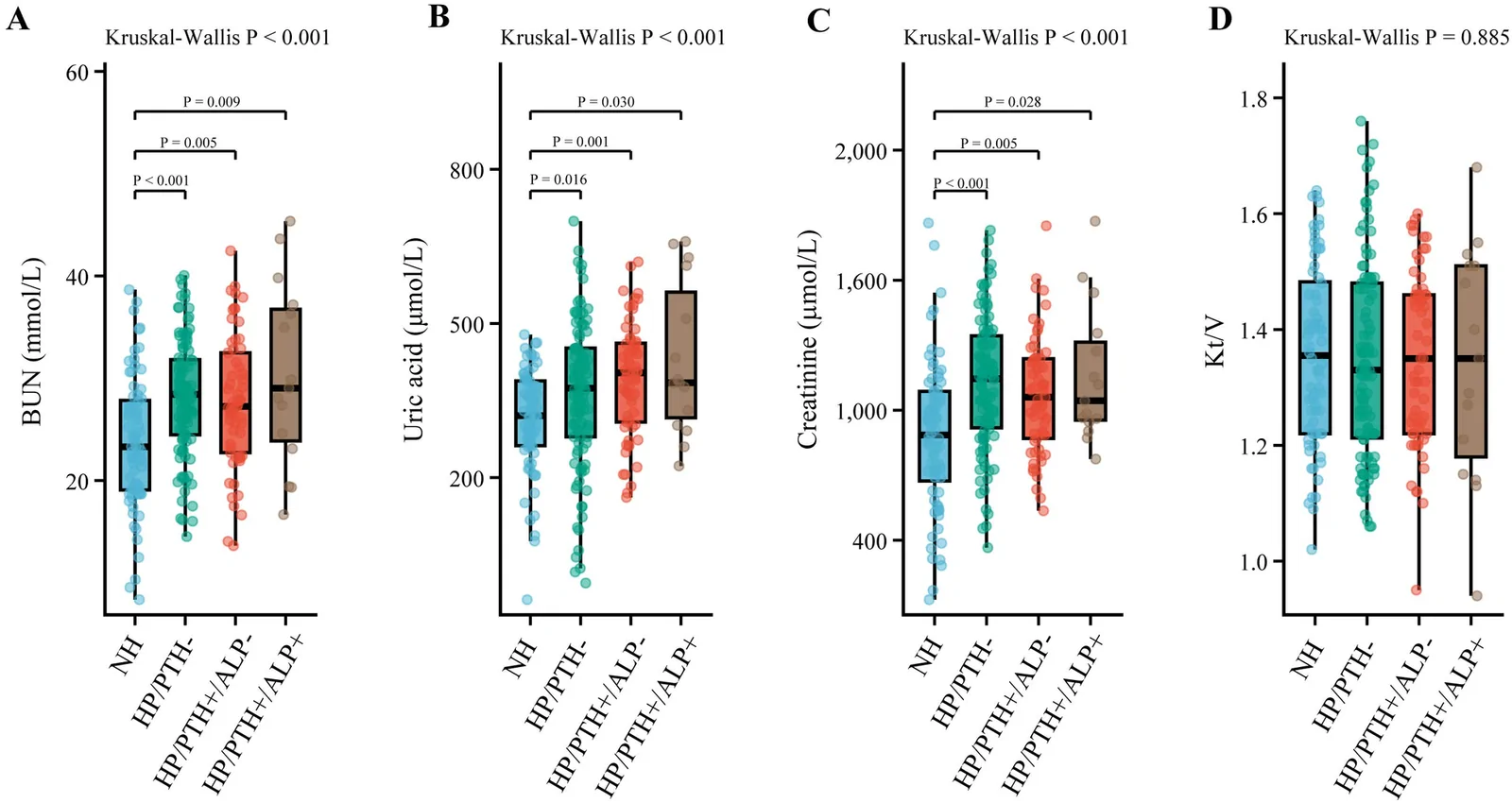
Comparison of non-defining biochemical and dialysis-related characteristics across predefined hyperphosphatemia-related biochemical patterns. Distributions of blood urea nitrogen (BUN) **(A)**, uric acid **(B)**, creatinine **(C)**, and Kt/V **(D)** are shown across the four predefined biochemical patterns. Overall group differences were assessed using the Kruskal–Wallis test. When the overall test was significant, pairwise comparisons were performed using Dunn's test with Holm adjustment for multiple comparisons; the *P* values shown above the brackets are Holm-adjusted values. NH, non-hyperphosphatemia; HP/PTH−, hyperphosphatemia without elevated parathyroid hormone; HP/PTH+/ALP−, hyperphosphatemia with elevated parathyroid hormone and alkaline phosphatase ≤125 U/L; HP/PTH+/ALP+, hyperphosphatemia with elevated parathyroid hormone and alkaline phosphatase >125 U/L; PTH, parathyroid hormone; ALP, alkaline phosphatase.

Serum phosphorus, PTH, and ALP were summarized to show the laboratory distributions underlying the predefined classification (Supplementary Figure S1). As expected from the threshold-based definitions, phosphorus values in the three hyperphosphatemic groups were above the 1.78 mmol/L cutoff, PTH was higher in the two PTH-positive groups, and ALP exceeded 125 U/L in the HP/PTH+/ALP + group. Treatment exposure was more frequent in the hyperphosphatemic groups, particularly for phosphate binders and active vitamin D. As shown in Supplementary Figure S2, Phosphate binder use was recorded in 14/72 patients (19.4%) in NH, compared with 104/106 (98.1%), 60/61 (98.4%), and 15/15 (100.0%) in the three hyperphosphatemic groups (*P* < 0.001). Active vitamin D use was 80.6%, 99.1%, 98.4%, and 100.0%, respectively (*P* < 0.001). Calcimimetic exposure ranged from 80.6% in NH to 100.0% in HP/PTH+/ALP + and did not differ significantly across groups (*P* = 0.153). These treatment data were considered descriptive because treatment indication and biochemical severity cannot be separated in this cross-sectional analysis.

### 3.3 Univariable associations with hyperphosphatemia

All univariable logistic regression models included the full analytical cohort of 254 patients, including 182 with hyperphosphatemia (Table 2). Older age was associated with lower odds of hyperphosphatemia, with an OR of 0.80 per 10-year increase (95% CI 0.65–0.98, *P* = 0.035). Among the biochemical measures, higher BUN, uric acid, and creatinine were each associated with higher odds of hyperphosphatemia. The corresponding ORs were 1.77 per 5 mmol/L increase in BUN (95% CI 1.39–2.24, *P* < 0.001), 1.63 per 100 μmol/L increase in uric acid (95% CI 1.24–2.13, *P* < 0.001), and 1.34 per 100 μmol/L increase in creatinine (95% CI 1.19–1.51, *P* < 0.001). Carbon dioxide combining power showed an inverse association (OR 0.90 per 1 mmol/L increase, 95% CI 0.82–1.00, *P* = 0.044). No clear univariable association was observed for sex, dialysis vintage, primary kidney disease, hemoglobin, serum iron, ferritin, transferrin saturation, calcium, lipid measures, Kt/V, dialysis frequency, dialysis-session duration, or dialysate calcium concentration (*P* > 0.05). Albumin showed a borderline association but did not reach the prespecified significance threshold (OR 1.50 per 5 g/L increase, 95% CI 0.98–2.29, *P* = 0.062). Treatment exposure was examined separately as a descriptive measure of CKD-MBD management and was not included in the association models. Phosphorus, PTH, and ALP were likewise not modeled as candidate correlates because they formed part of the prespecified biochemical classification.

**Table 2:** Univariable associations of clinical, biochemical, and dialysis-related factors with hyperphosphatemia.

Variable	OR (95% CI)	*P* value
***Demographic and clinical characteristics***		
Age, per 10 years	0.797 (0.645–0.984)	0.035
Sex, female vs. male	0.818 (0.471–1.421)	0.476
Dialysis vintage, per 12 months	0.980 (0.922–1.041)	0.506
Primary kidney disease	—	0.585
Diabetic nephropathy vs. primary glomerular disease	0.732 (0.370–1.448)	0.370
Hypertensive kidney damage vs. primary glomerular disease	1.674 (0.449–6.244)	0.443
Other causes vs. primary glomerular disease	1.087 (0.543–2.176)	0.815
***Biochemical characteristics***		
Hemoglobin, per 10 g/L	1.133 (0.940–1.366)	0.190
Albumin, per 5 g/L	1.497 (0.981–2.286)	0.062
Blood urea nitrogen, per 5 mmol/L	1.766 (1.390–2.243)	<0.001
Uric acid, per 100 μmol/L	1.628 (1.242–2.134)	<0.001
Creatinine, per 100 μmol/L	1.341 (1.191–1.511)	<0.001
Serum iron, per 5 μmol/L	1.194 (0.857–1.664)	0.294
Ferritin, per 100 ng/mL	1.030 (0.874–1.213)	0.728
Transferrin saturation, per 5%	0.975 (0.926–1.027)	0.343
Calcium, per 0.1 mmol/L	1.079 (0.945–1.232)	0.261
Triglyceride, per 1 mmol/L	0.994 (0.822–1.203)	0.953
Total cholesterol, per 1 mmol/L	0.878 (0.668–1.154)	0.351
LDL-C, per 1 mmol/L	0.975 (0.693–1.371)	0.883
HDL-C, per 1 mmol/L	1.040 (0.676–1.600)	0.858
CO2 combining power, per 1 mmol/L	0.904 (0.819–0.997)	0.044
***Dialysis-related characteristics***		
Kt/V, per 0.1	0.962 (0.814–1.136)	0.646
Dialysis frequency, 3 vs. 2 times/week	0.371 (0.082–1.689)	0.200
Dialysis duration, per 0.5 h	0.599 (0.175–2.051)	0.415
Dialysate calcium, 1.50 vs. 1.25 mmol/L	0.444 (0.096–2.055)	0.299

ORs and 95% CIs were estimated using univariable logistic regression, with hyperphosphatemia defined as serum phosphorus >1.78 mmol/L. All variables included in this table had complete data; each model used all 254 patients (182 with and 72 without hyperphosphatemia). Continuous variables were modeled using the increments shown in the table. For primary kidney disease, the *P* value on the main row is the overall likelihood-ratio test; *P* values for individual contrasts are Wald tests. Dialysis duration was modeled continuously per 0.5-h increment because the 3.0-h category contained only one patient; categorical modeling produced separation and unstable category-specific estimates. Phosphorus, PTH, and ALP were not included as candidate non-defining correlates. Treatment exposure variables were also excluded from this association table because of substantial potential for confounding by indication. These estimates describe cross-sectional associations and should not be interpreted as causal or predictive effects. OR, odds ratio; CI, confidence interval; PTH, parathyroid hormone; ALP, alkaline phosphatase; LDL-C, low-density lipoprotein cholesterol; HDL-C, high-density lipoprotein cholesterol.

### 3.4 Multivariable associations and assessment of nonlinear relationships

The functional form of BUN, uric acid, and creatinine was examined before finalizing the multivariable model. Restricted cubic spline analysis provided little evidence of nonlinearity for BUN (*P* for nonlinearity=0.766) or creatinine (*P* for nonlinearity=0.119). The overall association was borderline for BUN (*P* = 0.053) and significant for creatinine (*P* = 0.004). Uric acid showed an overall association with hyperphosphatemia (*P* = 0.005), with evidence of nonlinearity (*P* for nonlinearity=0.004) (Figure 4). The fitted curve suggested little change in adjusted odds across the lower and middle uric acid range, followed by an increase toward higher concentrations. The confidence bands widened substantially toward the extremes of the uric acid distribution, indicating reduced precision in these regions. BUN and creatinine were therefore retained as linear terms, whereas uric acid was modeled using the restricted cubic spline in the final model.

**Figure 4:**
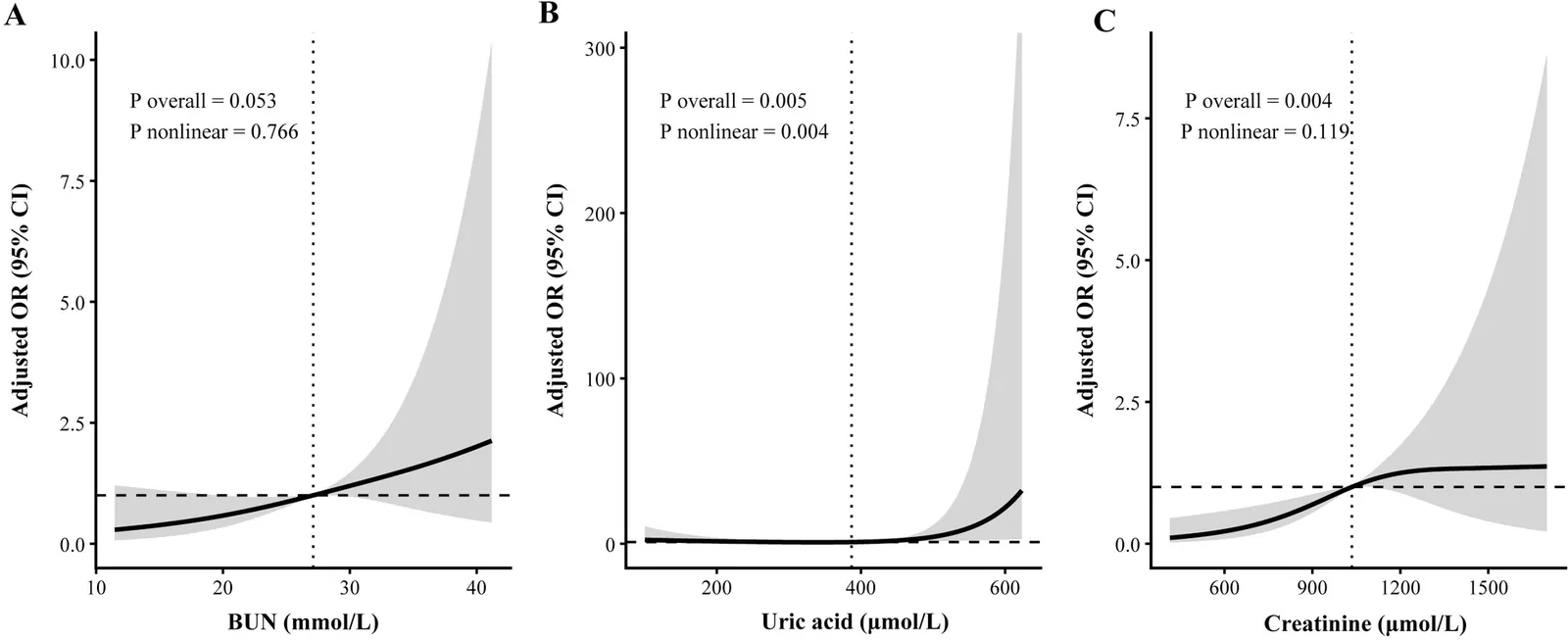
Restricted cubic spline analyses of the associations between selected biochemical markers and hyperphosphatemia. Restricted cubic spline models were used to examine the adjusted associations of blood urea nitrogen (BUN) **(A)**, uric acid **(B)**, and creatinine **(C)** with hyperphosphatemia. Three knots were placed at the 10th, 50th, and 90th percentiles of each marker. Solid lines represent adjusted odds ratios (ORs), and shaded areas indicate 95% confidence intervals. The horizontal dashed line denotes an OR of 1.0, and the vertical dotted line indicates the median value used as the reference. Each spline model was adjusted for age, sex, dialysis vintage, primary kidney disease, and the other biochemical and dialysis-related variables included in the primary multivariable model. *P* values for the overall association and for nonlinearity were obtained from likelihood-ratio tests.

After simultaneous adjustment for age, sex, dialysis vintage, primary kidney disease, BUN, uric acid, creatinine, and Kt/V, creatinine remained positively associated with hyperphosphatemia (OR 1.27 per 100 μmol/L increase, 95% CI 1.07–1.50, *P* = 0.005). The association with BUN was weaker after adjustment and did not meet the conventional significance threshold (OR 1.33 per 5 mmol/L increase, 95% CI 1.00–1.78, *P* = 0.054). The nonlinear association with uric acid remained evident in the adjusted model (overall *P* = 0.005; *P* for nonlinearity=0.004). Relative to the median uric acid concentration of 387.0 μmol/L, the adjusted OR was 1.14 (95% CI 0.64–2.05) at the 10th percentile (254.3 μmol/L) and 5.56 (95% CI 1.67–18.54) at the 90th percentile (517.7 μmol/L). The wide confidence interval for the upper-percentile contrast indicates substantial uncertainty in the magnitude of the association at high uric acid concentrations, and this estimate was therefore interpreted as exploratory. Age, sex, dialysis vintage, primary kidney disease, and Kt/V showed no clear adjusted association with hyperphosphatemia (*P* > 0.05) (Table 3; Figure 5). Multicollinearity was assessed using the corresponding linearized model, in which uric acid was entered as a single linear term rather than as restricted cubic spline basis functions; the maximum adjusted generalized variance inflation factor was 1.35.

**Table 3:** Multivariable associations of selected clinical, biochemical, and dialysis-related factors with hyperphosphatemia.

Variable	Adjusted OR (95% CI)	*P* value
Age, per 10 years	1.03 (0.79–1.33)	0.839
Female vs. Male	1.26 (0.64–2.46)	0.506
Dialysis vintage, per 12 months	0.95 (0.89–1.03)	0.217
**Primary kidney disease**		0.552
Diabetic nephropathy vs. primary glomerular disease	1.03 (0.44–2.43)	0.942
Hypertensive kidney damage vs. primary glomerular disease	1.52 (0.34–6.87)	0.586
Other causes vs. primary glomerular disease	1.70 (0.75–3.82)	0.201
Blood urea nitrogen, per 5 mmol/L	1.33 (1.00–1.78)	0.054
**Uric acid, restricted cubic spline**	Spline model	0.005
*P* for nonlinearity		0.004
Creatinine, per 100 μmol/L	1.27 (1.07–1.50)	0.005
Kt/V, per 0.1	0.96 (0.79–1.17)	0.714

Adjusted odds ratios (ORs) and 95% confidence intervals (CIs) were estimated using multivariable logistic regression, with hyperphosphatemia defined as serum phosphorus >1.78 mmol/L. Age, sex, dialysis vintage, primary kidney disease, blood urea nitrogen, uric acid, creatinine, and Kt/V were entered simultaneously into the model without stepwise or *P*-value-based variable selection. Blood urea nitrogen and creatinine were modeled as linear terms, whereas uric acid was modeled using a three-knot restricted cubic spline with knots at the 10th, 50th, and 90th percentiles because evidence of nonlinearity was observed. Accordingly, a single OR is not reported for uric acid; the reported *P* values represent the overall spline association and the test for nonlinearity, respectively. Male sex and primary glomerular disease served as the reference categories. The overall *P* value for primary kidney disease was obtained from a likelihood-ratio test, whereas *P* values for individual regression coefficients were based on Wald tests. All 254 patients were included in the final model. In the corresponding linearized model used to assess multicollinearity, the maximum adjusted generalized variance inflation factor was 1.35. These estimates reflect cross-sectional associations and should not be interpreted as causal or predictive effects. OR, odds ratio; CI, confidence interval; PTH, parathyroid hormone; ALP, alkaline phosphatase.

**Figure 5:**
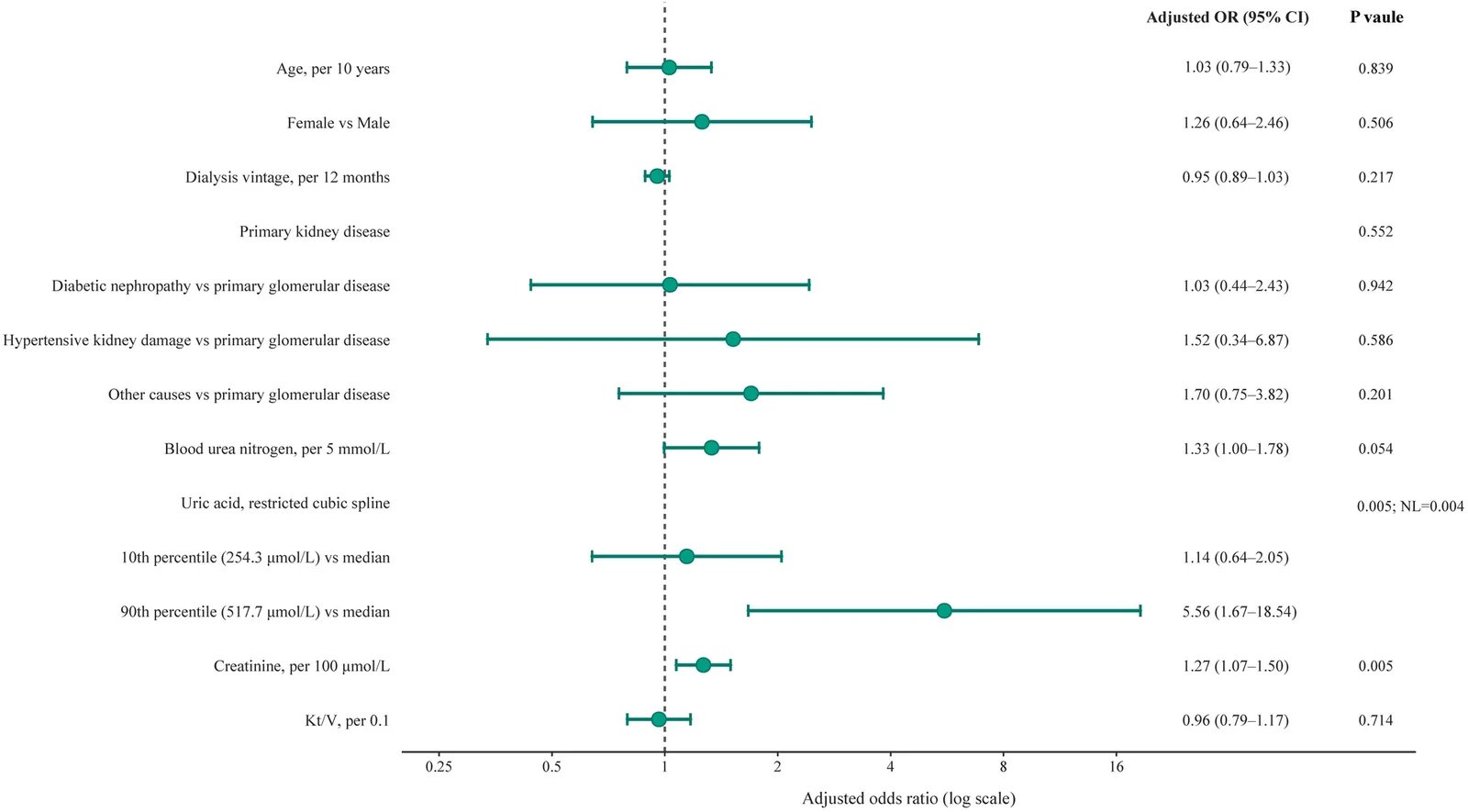
Forest plot of adjusted associations with hyperphosphatemia in the final multivariable logistic regression model. Points represent adjusted odds ratios (ORs), and horizontal lines indicate 95% confidence intervals (CIs); the vertical dashed line denotes an OR of 1.00. Age, sex, dialysis vintage, primary kidney disease, blood urea nitrogen, uric acid, creatinine, and Kt/V were entered simultaneously into the model. Primary glomerular disease and male sex were used as the reference categories. Uric acid was modeled using a three-knot restricted cubic spline with knots at 254.3, 387.0, and 517.7 μmol/L. Because a single OR does not adequately summarize a nonlinear association, adjusted ORs at the 10th and 90th percentiles are presented relative to the median (387.0 μmol/L). The overall *P* value for the uric acid spline was 0.005, with a *P* value for nonlinearity of 0.004. Overall *P* values for primary kidney disease and the uric acid spline were obtained using likelihood-ratio tests, whereas *P* values for individual regression coefficients were based on Wald tests. OR, odds ratio; CI, confidence interval; NL, *P* value for nonlinearity.

### 3.5 Sensitivity analyses and bootstrap robustness assessment

Using the stricter definition of hyperphosphatemia based on serum phosphorus >2.10 mmol/L, 122 of the 254 patients met the outcome definition. Creatinine was positively associated with hyperphosphatemia (OR 1.29 per 100 μmol/L increase, 95% CI 1.10–1.50, *P* = 0.002). The overall uric acid spline association remained evident (*P* = 0.008), with evidence of nonlinearity (*P* = 0.026). The adjusted OR at the 90th percentile of uric acid (517.7 μmol/L) relative to the median was 3.06 (95% CI 1.41–6.63). Although this estimate remained elevated, the confidence interval indicates appreciable uncertainty in the magnitude of the association at the upper end of the uric acid distribution. BUN was not significantly associated with hyperphosphatemia under this cutoff (OR 1.16 per 5 mmol/L increase, 95% CI 0.89–1.50, *P* = 0.280) (Supplementary Table S2). In the sensitivity analysis excluding the three records flagged during data-quality review, 251 patients remained, including 180 with hyperphosphatemia. Creatinine remained positively associated with hyperphosphatemia (OR 1.27 per 100 μmol/L increase, 95% CI 1.07–1.50, *P* = 0.005). The uric acid spline was significant overall (*P* = 0.005), with evidence of nonlinearity (*P* = 0.004). At the 90th percentile of uric acid, the adjusted OR relative to the median was 5.64 (95% CI 1.68–18.97). The wide confidence interval again indicates limited precision for the high-range uric acid contrast. The association with BUN remained borderline (OR 1.33 per 5 mmol/L increase, 95% CI 0.99–1.79, *P* = 0.054). No clear adjusted associations were observed for age, sex, dialysis vintage, primary kidney disease, or Kt/V in this sensitivity model (Supplementary Table S2).

Bootstrap analysis based on 1,000 nonparametric resamples yielded median ORs of 1.36 for BUN, 1.28 for creatinine, and 6.29 for uric acid at the 90th percentile relative to the median (Supplementary Table S3). For the high-range uric acid contrast, the bootstrap median OR of 6.29 was accompanied by a wide 95% percentile CI of 2.53–23.86. All 1,000 bootstrap models converged, and the median bootstrap estimates for most model terms were broadly similar in direction and magnitude to the original estimates. This consistency should not be taken to imply uniformly precise estimation, particularly for uric acid at the upper end of its distribution. The hypertensive kidney damage contrast was an exception, showing a highly right-skewed bootstrap distribution and a very wide upper confidence limit, consistent with sparse-data instability in this subgroup. Model convergence alone was therefore not considered evidence of coefficient stability or predictive performance.

## 4 Discussion

Hyperphosphatemia remained common in this maintenance hemodialysis cohort despite the widespread use of dietary measures, phosphate binders, and dialysis-based phosphate removal (1, 7). Current CKD-MBD guidance recommends interpreting serum phosphate together with PTH and ALP rather than in isolation (1). Large observational studies have likewise shown that abnormalities in phosphorus and PTH frequently coexist in hemodialysis populations and may carry different clinical implications (4, 5). In the present study, hyperphosphatemia was observed in 71.7% of patients, and the HP/PTH− pattern was the most frequent of the four predefined biochemical patterns. Beyond the markers used to construct the classification, BUN, uric acid, and creatinine differed across the groups, whereas Kt/V was similar. In the adjusted analysis, creatinine showed a positive association with hyperphosphatemia, while uric acid showed a nonlinear association. BUN was less consistent after adjustment and under the stricter phosphorus threshold. These findings are best viewed as a structured description of biochemical heterogeneity in routine dialysis care rather than evidence of distinct disease phenotypes or causal metabolic pathways.

The distribution of the four biochemical patterns provides some context for the high prevalence of hyperphosphatemia in this cohort. HP/PTH− was the largest subgroup, indicating that elevated serum phosphorus often occurred without a concurrent PTH concentration above the prespecified 300 pg/mL threshold. Only 15 patients (5.9%) were classified as HP/PTH+/ALP+, and comparisons involving this group should therefore be interpreted cautiously. Previous observational work has shown that combinations of phosphorus and PTH abnormalities are associated with different risks in patients receiving hemodialysis, and higher ALP has been associated with fracture and mortality in larger dialysis cohorts (3–5, 10, 11). These findings support considering phosphorus alongside other CKD-MBD markers, but they do not establish the four patterns used here as biologically distinct entities. In addition, the fixed ALP cutoff of 125 U/L was used only as an operational stratification threshold. Because adult ALP reference intervals vary by age and sex, use of a single cutoff may have introduced some age- or sex-related misclassification of ALP status, particularly in women.

BUN showed one of the clearest crude differences across the biochemical patterns, but its association weakened after multivariable adjustment and was no longer significant when hyperphosphatemia was defined using the stricter phosphorus threshold. The present findings therefore do not support describing BUN as an independent or stable predictor of hyperphosphatemia. A more plausible interpretation is that BUN and serum phosphorus may share upstream determinants in some patients. Dietary protein intake is one possible link because protein and phosphorus are often consumed together. In a large hemodialysis cohort, higher normalized protein catabolic rate and higher PTH were both associated with a greater likelihood of hyperphosphatemia (6). Dietary phosphorus burden and the phosphorus-to-protein ratio have also been associated with outcomes in hemodialysis populations (12), while nutrition guidelines emphasize the difficulty of reducing phosphorus intake without compromising adequate protein intake (13). BUN itself, however, is not a marker of dietary phosphorus exposure. It is also influenced by protein catabolism, volume status, dialysis timing, residual kidney function, and dialysis clearance. Because dietary intake, nPCR, urine output, and residual clearance were not available in this study, the BUN finding should remain hypothesis-generating.

Creatinine showed a more consistent positive association with hyperphosphatemia across the primary and sensitivity analyses, and the bootstrap estimate was close to the original adjusted estimate. Even so, serum creatinine in maintenance hemodialysis has several determinants, including creatinine generation, muscle mass, residual kidney clearance, and dialysis removal (14). A higher serum creatinine concentration could therefore accompany greater endogenous solute generation, lower residual clearance, or both, without implying a direct role in phosphate regulation. Uric acid also warrants cautious interpretation. Its association with hyperphosphatemia was nonlinear, with relatively little change across the lower and middle range and a rise in adjusted odds at higher concentrations. Uric acid in hemodialysis is related to purine and protein intake and has been proposed as a nutritional marker in this setting (15). However, the confidence intervals widened substantially at the upper end of the uric acid distribution. The adjusted OR at the 90th percentile was imprecise, and the corresponding bootstrap interval remained wide. The bootstrap results therefore support broad consistency in direction and magnitude for several model terms, but they do not remove the uncertainty surrounding the high-range uric acid estimate. This finding should be treated as exploratory rather than as evidence of a precise threshold effect or specific metabolic mechanism.

The absence of a clear association between Kt/V and hyperphosphatemia should not be interpreted as evidence that dialysis prescription is unimportant for phosphate control. Kt/V reflects urea kinetics, whereas phosphate follows more complex multicompartment kinetics and undergoes substantial post-dialysis redistribution. Phosphate removal depends on treatment duration, treatment frequency, dialyzer characteristics, and blood and dialysate flow, among other factors (16, 17). Experimental and clinical kinetic studies have shown that urea clearance does not adequately represent phosphate removal (17). In the present cohort, dialysis prescription also showed limited variation, with most patients treated three times weekly for approximately 4 h per session. This restricted range reduces the ability of a cross-sectional analysis to detect prescription-related differences. Residual kidney function, blood and dialysate flow rates, membrane characteristics, ultrafiltration volume, and the long interdialytic interval were not consistently available, which further limits interpretation of the null Kt/V finding.

The treatment-exposure results should also remain descriptive. Phosphate binder and active vitamin D use were much more frequent in the hyperphosphatemic groups, whereas calcimimetic use was high across all four patterns. These therapies are commonly initiated or intensified because biochemical abnormalities are already present or difficult to control, making cross-sectional comparisons particularly vulnerable to confounding by indication. The observed treatment distribution therefore cannot establish treatment efficacy, treatment failure, or harm. Phosphate binders can lower serum phosphorus, although comparative evidence for patient-important outcomes remains less certain (18). Active vitamin D and calcimimetics are used mainly in the management of secondary hyperparathyroidism and can substantially alter biochemical indices (19, 20). Cinacalcet, for example, lowers PTH in hemodialysis patients, whereas the EVOLVE trial did not demonstrate a statistically significant reduction in its unadjusted primary cardiovascular composite outcome (21). Because treatment dose, duration, adherence, and longitudinal treatment changes were not consistently available, treatment exposure in this study is more appropriately interpreted as a marker of CKD-MBD management intensity than as an explanatory variable.

Several limitations should be considered when interpreting these findings. First, this was a single-center retrospective cross-sectional study, so temporality and causal direction cannot be established. No prospective sample-size calculation was performed because all potentially eligible patients within the study period were screened. The available number of events supported a deliberately limited fixed multivariable model, but the cohort size still constrained precision for sparse categorical contrasts and for the smallest biochemical subgroup. Second, 178 of the 483 initially eligible patients were excluded because one or more required clinical or laboratory variables were unavailable. These cases were not retained in the analytical dataset, and the source information needed to reconstruct variable-specific missingness or compare excluded and included patients was no longer retrievable from the original clinical and laboratory systems at the time of revision. Complete-case selection may therefore have affected both the observed prevalence of hyperphosphatemia and the estimated associations, but the direction and magnitude of this potential bias cannot be determined. Third, although the study used a defined index-assessment rule and serum phosphorus served as the anchor measurement, sampling conditions could not be verified uniformly across the cohort. Blood sampling was intended to occur before dialysis, but a measurement could only be classified as verifiably predialysis when the laboratory timestamp preceded the documented dialysis start time. For some records, this relationship remained uncertain. This is particularly important because phosphorus, BUN, creatinine, and uric acid can vary according to dialysis timing and the interdialytic interval. Because serum phosphorus was used to define both the binary hyperphosphatemia outcome and the biochemical patterns, uncertainty in sampling timing may have introduced not only measurement variability but also some degree of classification error. The direction and magnitude of any such misclassification cannot be determined from the available retrospective data. PTH and ALP were selected within predefined windows around the phosphorus anchor measurement rather than assumed to have been measured under identical conditions in every patient. Finally, several potentially relevant factors were unavailable, including dietary phosphorus and protein intake, nPCR, phosphate additives, residual kidney function and urine output, inflammatory markers, FGF23, 25-hydroxyvitamin D, bone-specific ALP, detailed dialysis prescription, and medication dose and adherence. These omissions limit mechanistic interpretation of the observed associations. The HP/PTH+/ALP + group included only 15 patients, and the use of a single ALP cutoff may have introduced additional classification uncertainty because adult reference limits vary by sex and age. The biochemical patterns were not evaluated in relation to fractures, vascular calcification, hospitalization, cardiovascular events, mortality, or longitudinal CKD-MBD control. Their reproducibility, prognostic value, and clinical usefulness therefore remain to be established in larger prospective cohorts with standardized predialysis sampling and repeated biochemical measurements.

## 5 Conclusion

Hyperphosphatemia was common in this MHD cohort and frequently occurred without concomitant PTH elevation, while only a small proportion of patients showed combined elevations of phosphorus, PTH, and ALP. Creatinine remained positively associated with hyperphosphatemia after adjustment, whereas uric acid showed a nonlinear association that was less precisely estimated at higher concentrations. The association with BUN was weaker and less consistent across sensitivity analyses. These findings do not support treatment decisions based on the four biochemical patterns, but they show that routinely available laboratory data may help describe clinically relevant heterogeneity beyond serum phosphorus alone. This simple biochemical framework may therefore be useful for generating testable questions about phosphate control in MHD. Further multicenter studies using standardized predialysis sampling, repeated measurements, and more complete dietary, dialysis, residual kidney function, and treatment data are needed to determine whether these patterns are reproducible and clinically informative.

## Data Availability

The raw data supporting the conclusions of this article will be made available by the authors, without undue reservation.
