# Supplementary material for: Hyperphosphatemia-related biochemical profiles and clinical correlates in patients receiving maintenance hemodialysis: a single-center retrospective cross-sectional study
Source: Front Med (Lausanne). 2026 Oct 28;13:1910971. doi: 10.3389/fmed.2026.1910971 (PMC13751438; doi:10.3389/fmed.2026.1910971)
Supplement: Supplementary file 1 [file Table4.docx]

Supplementary Material

# 1 Supplementary Figures

**Figure S1.** Distribution of the biochemical markers used to define the predefined hyperphosphatemia-related biochemical patterns. Serum phosphorus (A), parathyroid hormone (PTH) (B), and alkaline phosphatase (ALP) (C) are shown across the four predefined biochemical patterns. Dashed horizontal lines indicate the classification thresholds for serum phosphorus (1.78 mmol/L), PTH (300 pg/mL), and ALP (125 U/L), respectively. NH, non-hyperphosphatemia; HP/PTH−, hyperphosphatemia without elevated PTH; HP/PTH+/ALP−, hyperphosphatemia with elevated PTH and ALP ≤125 U/L; HP/PTH+/ALP+, hyperphosphatemia with elevated PTH and ALP >125 U/L.

**Figure S2.** Treatment exposure across predefined hyperphosphatemia-related biochemical patterns. The proportions of patients receiving phosphate binders (A), active vitamin D (B), and calcimimetics (C) are shown for each biochemical pattern. Values above the bars are presented as n/N (%), with percentages calculated from available exposure data. One patient in the HP/PTH− group had missing information on calcimimetic use. Treatment exposure is shown descriptively and should not be interpreted as evidence of treatment efficacy or harm because treatment selection may reflect underlying disease severity and clinical management. NH, non-hyperphosphatemia; HP/PTH−, hyperphosphatemia without elevated parathyroid hormone; HP/PTH+/ALP−, hyperphosphatemia with elevated parathyroid hormone and alkaline phosphatase ≤125 U/L; HP/PTH+/ALP+, hyperphosphatemia with elevated parathyroid hormone and alkaline phosphatase >125 U/L; PTH, parathyroid hormone; ALP, alkaline phosphatase.

# 2 Supplementary Tables

**Table S1.** Distribution of predefined hyperphosphatemia-related biochemical patterns according to sex, primary kidney disease, and dialysis vintage.

| **Variable** | **Category** | **NH N = 72** | **HP/PTH− N = 106** | **HP/PTH+/ALP− N = 61** | **HP/PTH+/ALP+ N = 15** | **Total** | **P value** |
| --- | --- | --- | --- | --- | --- | --- | --- |
| **Sex** | Male | 40 (26.7%) | 64 (42.7%) | 36 (24.0%) | 10 (6.7%) | 150 | 0.849 |
|  | Female | 32 (30.8%) | 42 (40.4%) | 25 (24.0%) | 5 (4.8%) | 104 |  |
| **Primary kidney disease** | Primary glomerular disease | 34 (27.9%) | 49 (40.2%) | 30 (24.6%) | 9 (7.4%) | 122 | 0.275 |
|  | Diabetic nephropathy | 19 (34.5%) | 24 (43.6%) | 10 (18.2%) | 2 (3.6%) | 55 |  |
|  | Hypertensive kidney damage | 3 (18.8%) | 8 (50.0%) | 2 (12.5%) | 3 (18.8%) | 16 |  |
|  | Other causes | 16 (26.2%) | 25 (41.0%) | 19 (31.1%) | 1 (1.6%) | 61 |  |
| **Dialysis vintage** | <36 months | 20 (28.2%) | 32 (45.1%) | 17 (23.9%) | 2 (2.8%) | 71 | 0.733 |
|  | 36–59 months | 16 (26.2%) | 28 (45.9%) | 12 (19.7%) | 5 (8.2%) | 61 |  |
|  | ≥60 months | 36 (29.5%) | 46 (37.7%) | 32 (26.2%) | 8 (6.6%) | 122 |  |

Values are presented as n (row %); percentages were calculated within each category. P values compare the distribution of the four predefined biochemical patterns across categories. Sex was assessed using Pearson’s chi-square test. Primary kidney disease and dialysis vintage were assessed using Fisher’s exact test with Monte Carlo simulation (200,000 replicates) because of sparse expected cell counts. NH, serum phosphorus ≤1.78 mmol/L; HP/PTH−, serum phosphorus >1.78 mmol/L and PTH ≤300 pg/mL (ALP was not used for assignment to this group); HP/PTH+/ALP−, serum phosphorus >1.78 mmol/L, PTH >300 pg/mL, and ALP ≤125 U/L; HP/PTH+/ALP+, serum phosphorus >1.78 mmol/L, PTH >300 pg/mL, and ALP >125 U/L. NH, non-hyperphosphatemia; PTH, parathyroid hormone; ALP, alkaline phosphatase.

**Table S2.** Sensitivity analyses of estimates from the final multivariable model for hyperphosphatemia.

|  | **Primary analysis Phosphorus >1.78 mmol/L; N=254, events=182** | | **Sensitivity analysis 1 Phosphorus >2.10 mmol/L; N=254, events=122** | | **Sensitivity analysis 2 QC exclusions; N=251, events=180** | |
| --- | --- | --- | --- | --- | --- | --- |
| **Variable** | **Adjusted OR (95% CI)** | **P value** | **Adjusted OR (95% CI)** | **P value** | **Adjusted OR (95% CI)** | **P value** |
| Age, per 10 years | 1.03 (0.79–1.33) | 0.839 | 0.90 (0.71–1.15) | 0.404 | 1.04 (0.80–1.35) | 0.783 |
| Female vs Male | 1.26 (0.64–2.46) | 0.506 | 1.32 (0.71–2.46) | 0.387 | 1.29 (0.66–2.55) | 0.455 |
| Dialysis vintage, per 12 months | 0.95 (0.89–1.03) | 0.217 | 0.97 (0.91–1.04) | 0.435 | 0.97 (0.90–1.04) | 0.381 |
| **Primary kidney disease** |  | 0.552 |  | 0.680 |  | 0.571 |
| Diabetic nephropathy vs primary glomerular disease | 1.03 (0.44–2.43) | 0.942 | 1.10 (0.49–2.46) | 0.813 | 1.07 (0.45–2.53) | 0.873 |
| Hypertensive kidney damage vs primary glomerular disease | 1.52 (0.34–6.87) | 0.586 | 2.00 (0.56–7.09) | 0.285 | 1.60 (0.35–7.28) | 0.542 |
| Other causes vs primary glomerular disease | 1.70 (0.75–3.82) | 0.201 | 0.92 (0.44–1.90) | 0.813 | 1.69 (0.75–3.83) | 0.205 |
| Blood urea nitrogen, per 5 mmol/L | 1.33 (1.00–1.78) | 0.054 | 1.16 (0.89–1.50) | 0.280 | 1.33 (0.99–1.79) | 0.054 |
| **Uric acid, restricted cubic spline** |  | 0.005 |  | 0.008 |  | 0.005 |
| P for nonlinearity |  | 0.004 |  | 0.026 |  | 0.004 |
| 10th percentile (254.3 μmol/L) vs median | 1.14 (0.64–2.05) |  | 0.96 (0.58–1.60) |  | 1.14 (0.64–2.03) |  |
| 90th percentile (517.7 μmol/L) vs median | 5.56 (1.67–18.54) |  | 3.06 (1.41–6.63) |  | 5.64 (1.68–18.97) |  |
| Creatinine, per 100 μmol/L | 1.27 (1.07–1.50) | 0.005 | 1.29 (1.10–1.50) | 0.002 | 1.27 (1.07–1.50) | 0.005 |
| Kt/V, per 0.1 | 0.96 (0.79–1.17) | 0.714 | 1.01 (0.85–1.21) | 0.891 | 0.96 (0.79–1.16) | 0.647 |

The same multivariable logistic regression model was used in the primary and sensitivity analyses, with age, sex, dialysis vintage, primary kidney disease, blood urea nitrogen, uric acid, creatinine, and Kt/V entered simultaneously. No stepwise or P-value-driven variable selection was performed. Hyperphosphatemia was defined as serum phosphorus >1.78 mmol/L in the primary analysis. Sensitivity analysis 1 applied a stricter cutoff of serum phosphorus >2.10 mmol/L, whereas sensitivity analysis 2 repeated the primary analysis after exclusion of three records flagged during data-quality review. Because the association between uric acid and hyperphosphatemia was modeled nonlinearly, a single OR was not reported; instead, the overall spline P value, the P value for nonlinearity, and adjusted ORs at the 10th and 90th percentiles relative to the median are presented. Overall P values for primary kidney disease and the uric acid spline were obtained using likelihood-ratio tests, whereas P values for individual regression coefficients were based on Wald tests. OR, odds ratio; CI, confidence interval; QC, quality control.

**Table S3.** Bootstrap robustness assessment of estimates from the final multivariable model for hyperphosphatemia.

| **Variable** | **Original adjusted OR (95% CI)** | **Bootstrap median OR (95% percentile CI)** | **Bias, log(OR)** | **Usable bootstrap estimates, n** |
| --- | --- | --- | --- | --- |
| Age, per 10 years | 1.03 (0.79–1.33) | 1.04 (0.78–1.37) | 0.011 | 1000 |
| Female vs Male | 1.26 (0.64–2.46) | 1.23 (0.59–2.81) | -0.002 | 1000 |
| Dialysis vintage, per 12 months | 0.95 (0.89–1.03) | 0.95 (0.88–1.03) | -0.003 | 1000 |
| Diabetic nephropathy vs primary glomerular disease | 1.03 (0.44–2.43) | 1.02 (0.43–2.61) | -0.015 | 1000 |
| Hypertensive kidney damage vs primary glomerular disease† | 1.52 (0.34–6.87) | 1.66 (0.43–8.31e+06) | 0.798 | 1000 |
| Other causes vs primary glomerular disease | 1.70 (0.75–3.82) | 1.75 (0.78–4.46) | 0.037 | 1000 |
| Blood urea nitrogen, per 5 mmol/L | 1.33 (1.00–1.78) | 1.36 (1.00–1.93) | 0.022 | 1000 |
| Uric acid at 10th percentile (254.3 μmol/L) vs median | 1.14 (0.64–2.05) | 1.15 (0.63–2.68) | 0.050 | 1000 |
| Uric acid at 90th percentile (517.7 μmol/L) vs median | 5.56 (1.67–18.54) | 6.29 (2.53–23.86) | 0.179 | 1000 |
| Creatinine, per 100 μmol/L | 1.27 (1.07–1.50) | 1.28 (1.07–1.53) | 0.009 | 1000 |
| Kt/V, per 0.1 | 0.96 (0.79–1.17) | 0.96 (0.78–1.20) | -0.002 | 1000 |

Bootstrap stability was assessed using 1000 nonparametric case-resampling replicates of the final multivariable logistic regression model. The original model specification was retained in every replicate, and no variable selection was performed. Uric acid was modeled using the same three-knot restricted cubic spline as in the primary analysis, with fixed knots at 254.3, 387.0, and 517.7 μmol/L. Because a single OR cannot summarize a nonlinear association, spline effects are shown at the 10th and 90th percentiles relative to the median. Bootstrap 95% confidence intervals are percentile-based. Bias is the difference between the mean bootstrap coefficient and the original coefficient on the log-odds scale. †The bootstrap distribution for the hypertensive kidney damage contrast was extremely right-skewed, producing a very wide upper confidence limit. This reflects sparse-data instability in resampled datasets and should not be interpreted as a precise effect estimate. Model convergence alone does not imply coefficient stability. 1000 of 1000 bootstrap models converged. OR, odds ratio; CI, confidence interval.
